# Supplementary material for: The anatomy of unfolding of Yfh1 is revealed by site-specific fold stability analysis measured by 2D NMR spectroscopy
Source: Commun Chem. 2021 Sep 6;4:127. doi: 10.1038/s42004-021-00566-3 (PMC7612453; doi:10.1038/s42004-021-00566-3)
Supplement: Supplementary file 2 — Supporting Information [file 42004_2021_566_MOESM2_ESM.pdf]

Supporting Information

The anatomy of unfolding of Yfh1 is revealed by site-specific fold stability analysis measured by 2D NMR spectroscopy

Rita Puglisi[1], Gogulan Karunanithy[2], D. Flemming Hansen[2], Annalisa Pastore[1]*, Piero Andrea Temussi[1]*

[1]UK-DRI at King's College London, The Wohl Institute, 5 Cutcombe Rd, SE59RT London (UK)

[2]Department of Structural Biology, Division of Biosciences, University College London, London, UK, WC1E 6BT

Simulations to estimate the accuracy of the derived stability curves

As input for the least-squares fitting of the stability curves (eq. (3)), the residue-specific populations of the folded species as a function of temperature, f_f(T), were derived directly from the peak-intensities observed in unenhanced 2D ^{15}N-^{1}H HSQC spectra. Since the folded state of Yhf1 is in a chemical equilibrium with the unfolded state, the folding reaction needs to be in the slow-exchange regime (McConnell, 1958) on the NMR time scale for intensities to strictly be proportional with populations.

During the two INEPT periods of the unenhanced ^{15}N-^{1}H HSQC experiment, chemical exchange of transverse single-quantum ^{1}H coherences, $H_{x,y}$ and $2H_{x,y}N_z$, between the folded and unfolded species leads to line-broadening and therefore to a loss of intensity. The effect of this line-broadening on the derived populations, f_f(T), and thus on the derived stability curves, was estimated by simulating a two-site exchanging system with the Liouvillian evolution matrix (Allard et al., 1998):

$$\mathbf{\Gamma} = \begin{pmatrix} -R_{2,f} - k_{fu} & k_{uf} \\ k_{fu} & -R_{2,u} - k_{uf} - i\Delta\omega_{fu} \end{pmatrix} \quad (1)$$

where

$$\begin{pmatrix} M_f \\ M_u \end{pmatrix}(t) = \exp(\mathbf{\Gamma}\, t)\begin{pmatrix} M_f \\ M_u \end{pmatrix}(0) \quad (2)$$

and $R_{2,f}$ and $R_{2,u}$ are the single-quantum proton transverse relaxation rates in the folded and unfolded species, respectively; $\Delta\omega_{fu}$ is the proton chemical shift difference between the folded and unfolded state (rad/sec); k_{fu} and k_{uf} are the rate constants for the unfolding and folding

reactions, respectively; and M_f and M_u represent the (complex) transverse magnetisations in the folded and unfolded states. The one-bond ^{1}H-^{15}N scalar-coupling evolution was neglected.

The chemical shift differences, $\Delta\omega_{fu}$, were calculated under the assumption that the chemical shifts of the unfolded state are those of a random-coil. Therefore, from the chemical shift assignment of the folded state and based on predicted random-coil chemical shifts (Wishart et al., 1995) for the unfolded state, $\Delta\omega_{fu}$ values were calculated for all residues for which a chemical shift assignment was available. The rate constants for the folding and unfolding reactions, k_{fu} and k_{uf}, were derived from Figure 4 of Bonetti et al. (2014), where 30 points for k_{fu} and k_{uf} were extracted in the temperature range from 271 K to 340 K. Subsequently, a quadratic spline interpolation (*scipy.interpolate.interp1d*) was used to calculate the rate constants at any given temperature between 271 K and 340 K.

The intrinsic transverse relaxation rates were estimated by first calculating the rotational correlation time, τ_R, for the folded protein over the temperature range 278–313 K. Based on previous ^{15}N relaxation studies on another frataxin ortholog (Rasheed et al., 2019) and on the size of Yhf1 (Maciejewski et al., 2000), it was assumed that the rotational correlation time is 6 ns at 298 K. Using known relationships between the viscosity, η, and temperature for H_2O and D_2O (Nagashima et al., 1977), the rotational correlation time was extrapolated from 298 K to the desired temperature using the relationship $\tau_R \propto \eta(T)/T$. This led to τ_R in the range from 11 ns to 4.2 ns for temperatures in the range from 278–313 K.

Subsequently, $R_{2,f}$ and $R_{2,u}$ were calculated using a numerical integration of the interaction Hamiltonians following standard Bloembergen-Purcell-Pound theory (Bloembergen et al., 1948; Abragam, 1961). These interactions included: ^{1}H^N-^{15}N dipole-dipole interaction, ^{1}H^N chemical shift anisotropy, ^{15}N chemical shift anisotropy, and dipole-dipole interaction between ^{1}H^N and an external proton placed 1.85 Å from the ^{1}H^N spin. For short delays, as those in the INEPT of ^{15}N-^{1}H HSQC experiments, the interaction with remote ^{1}H spins can be approximated by a single proton at a distance of 1.85 Å (Hansen et al., 2007). Furthermore, a ^{1}H-^{15}N distance of 1.02 Å, a ^{1}H CSA of 10 ppm and a ^{15}N CSA of -172 ppm were assumed. The spectral density function, $J(\omega)$, was modelled within the model-free approach (Lipari and Szabo, 1982), where:

$$J(\omega) = \frac{2}{5}\frac{S^2\tau_R}{1+\omega^2\tau_R^2} + \frac{2}{5}\frac{(1-S^2)\tau_e}{1+\omega^2\tau_e^2} \quad (3)$$

In all cases a fast internal librational motion with a time-constant of τ_e = 80 ps was assumed. Order parameters, S^2, of 0.8 and 0.15 were assumed for the folded and unfolded states

respectively. This calculation led to a R_{2f} between 30 s^{-1} and 12 s^{-1} and an intrinsic R_2 for the unfolded state, $R_{2,u,in}$, between 6.5 and 3.1 s^{-1} over the temperature range 278–313 K.

Hydrogen exchange was incorporated by assuming high protection factors in the folded state. Thus, the hydrogen-exchange rate, k_{HDX}, is much smaller than the intrinsic transverse relaxation rate, $k_{HDX} << R_{2,f}$, and the hydrogen exchange within the folded state can be neglected. For the unfolded state, which was assumed to be a random-coil, hydrogen exchange rates were calculated using the SPHERE web-application (Yu-Zhu Zhang, PhD thesis; Bai 1993). Finally, the $R_{2,u}$ included in the calculation of the Liouvillian, $\mathbf{\Gamma}$, was calculated as: $R_{2,u} = R_{2u,in} + k_{HDX}$.

The populations derived from the experimental ^{15}N-^{1}H HSQC spectra, f_f and f_u, were used as input to the simulation, with

$$\begin{pmatrix} M_f \\ M_u \end{pmatrix} (0) = \begin{pmatrix} f_f \\ f_u \end{pmatrix} \qquad (4)$$

These magnetisations were propagated through the two INEPTs of the unenhanced HSQC, each described by the evolution matrix, $\exp(\mathbf{\Gamma}\,\tau)\exp(\mathbf{\Gamma}^{\dagger}\,\tau)$, with $\tau = 2.3$ ms. Finally, the value of M_f was used as a proxy for the measured intensity of the cross-peak corresponding to the folded state. The simulation was repeated for 15 temperatures in the range 278–313 K and the stability curve, $\Delta G(T)$, calculated as for the experimental populations.

References

Abragam, A. The Principles of Nuclear Magnetism. Clarendon Press, 1961.

Allard, P., Helgstrand, M., Hard, T. The complete homogeneous master equation for a heteronuclear two-spin system in the basis of Cartesian product operators. J Magn. Reason. 134:7–16, 1998, doi: 10.1006/jmre.1998.1509.

Bai Y, Milne JS, Mayne L, Englander SW. Primary structure effects on peptide group hydrogen exchange. Proteins. 17, 75-86, 1993, doi: 10.1002/prot.340170110.

Bloembergen, N., Purcell, E.M., Pound, R.V. Relaxation effects in nuclear magnetic resonance absorption Physical Review 73, 679-712, 1948.

Bonetti, D., Toto, A., Giri, R., Morrone, A., Sanfelice, D., Pastore, A., Temussi, P., Gianni, S. and Brunori, M. The kinetics of folding of frataxin. Phys. Chem. Chem. Phys., 16, 6391-7, 2014, doi: 10.1039/c3cp54055c.

Hansen DF, Yang D, Feng H, Zhou Z, Wiesner S, Bai Y, Kay LE. An exchange-free measure of 15N transverse relaxation: an NMR spectroscopy application to the study of a folding intermediate with pervasive chemical exchange. J Am Chem Soc. 2007 Sep 19;129(37):11468-79. doi: 10.1021/ja072717t.

Lipari, G.; Szabo, A. Model-free approach to the interpretation of nuclear magnetic resonance relaxation in macromolecules. 1. Theory and range of validity. J. Am. Chem. Soc. 104, 4546-4559, 1982, doi.org/10.1021/ja00381a009.

McConnell, H.M. Reaction Rates by Nuclear Magnetic Resonance J. Chem. Phys. 28, 430-431, 1958, doi: 10.1063/1.1744152.

Nagashima, A. Viscosity of water substance - new international formulation and its background. J. Phys. Chem. Ref. Data 6, 1133-1166, 1977.

Rasheed, M., Jamshidiha, M., Puglisi, R., Yan, R., Cota, E., Pastore, A. Structural and functional characterization of a frataxin from a thermophilic organism. FEBS J. 286, 495-506, 2019, doi: 10.1111/febs.14750.

Wishart, D.S., Bigam, C.G., Holm, A., Hodges, R.S., Sykes, B.D. (1)H, (13)C and (15)N random coil NMR chemical shifts of the common amino acids. I. Investigations of nearest-neighbor effects. J Biomol NMR. 5, 332, 1995. doi: 10.1007/BF00211764.

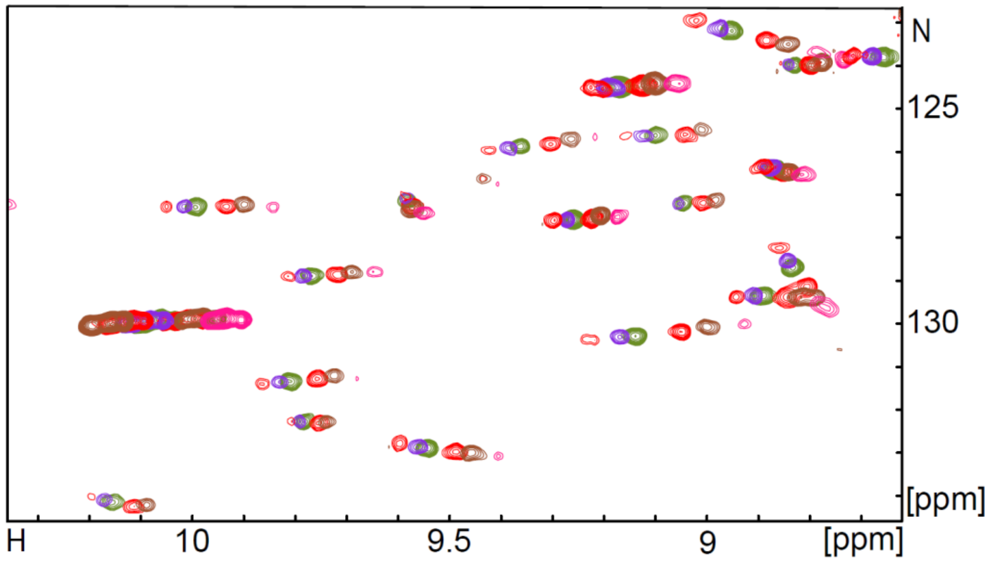

Figure S1. Representative examples of the raw data: superposition of HSQC spectra recorded at different temperatures from 280.5 K to 310.5 K in steps of 5 K. Typically, the resonances move downshift in the proton dimension as already noticed in Adrover et al., 2012.

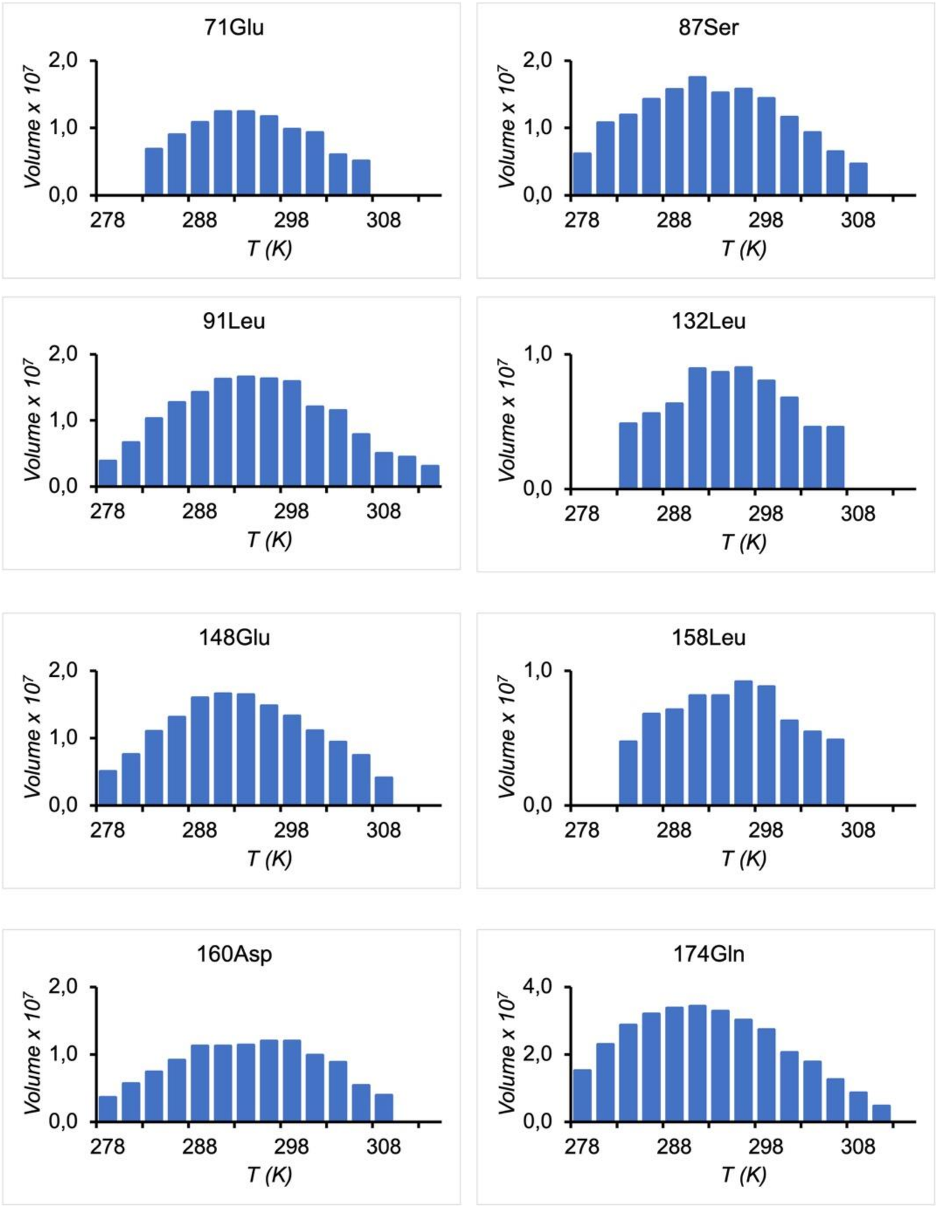

Figure S2. Typical plots of the dependence of residue volumes vs temperature for arbitrarily chosen residues. The volumes were quantified by summation of peak intensities in a set box using the CCPNMR software.

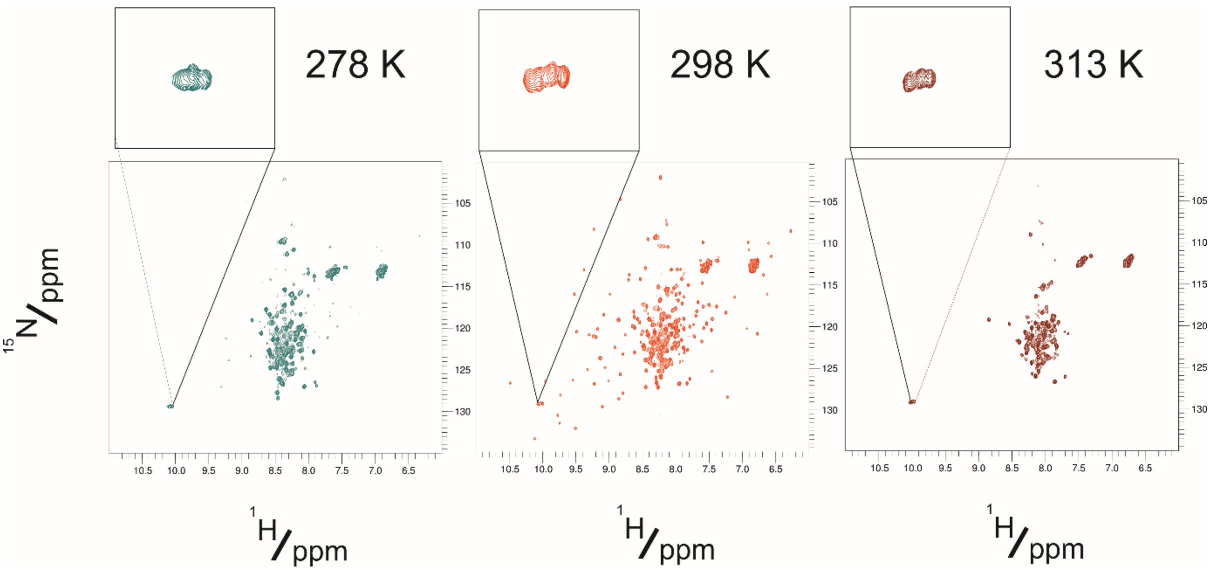

Figure S3. HSQC spectra of Yfh1 at 278 K, 298 K and 313 K. The spectra were collected at 700 MHz. The well distinguishable resonances of the Tryptophan indoles are zoomed to demonstrate that some peaks could be observed nearly at all temperatures in the range 278-313 K, while others disappeared.

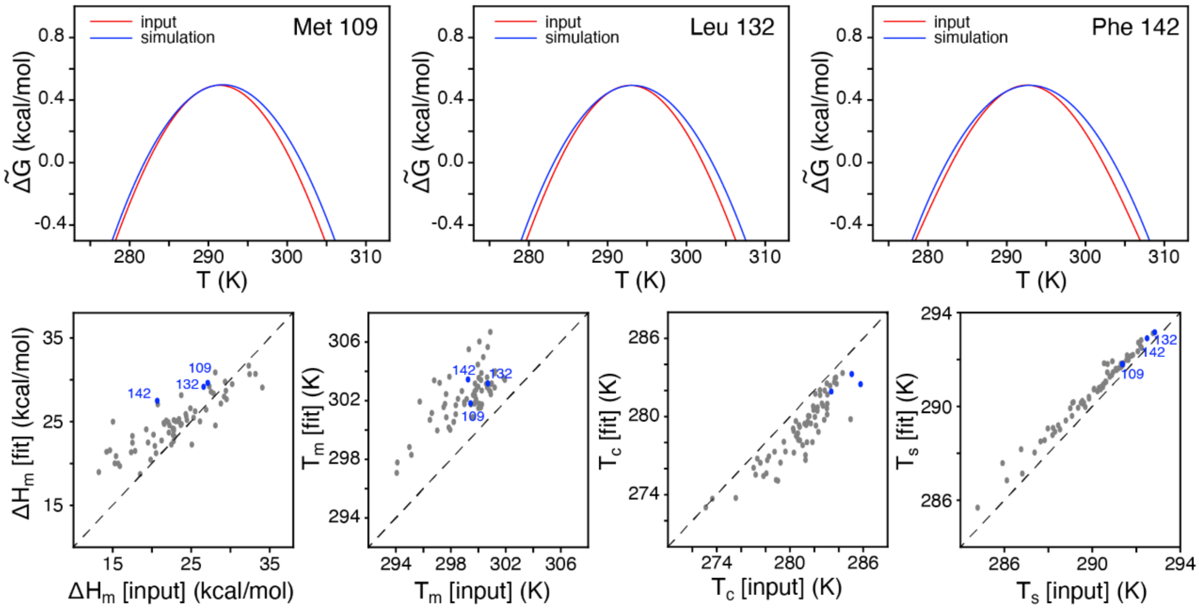

Figure S4 – Demonstration of the combined effect of three contributions: the exchange between folded and unfolded, the differential relaxation, and the hydrogen exchange. Top row: three representative examples. In red is the fit from NMR intensities and in blue is the fit obtained by taking into account the three contributions. Bottom row: Correlation between the thermodynamic parameters (ΔH_m, T_m, T_c, T_s) obtained from the fit of experimental NMR intensities (x-axis) and those obtained after added the three contributions and fitting the data again (y-axis). As such, the x-axis corresponds to the parameters from red curves shown in the

top row and the y-axis corresponds to the blue curves. For this protein, T_s, but not the other parameters, is well determined simply using the NMR intensities.

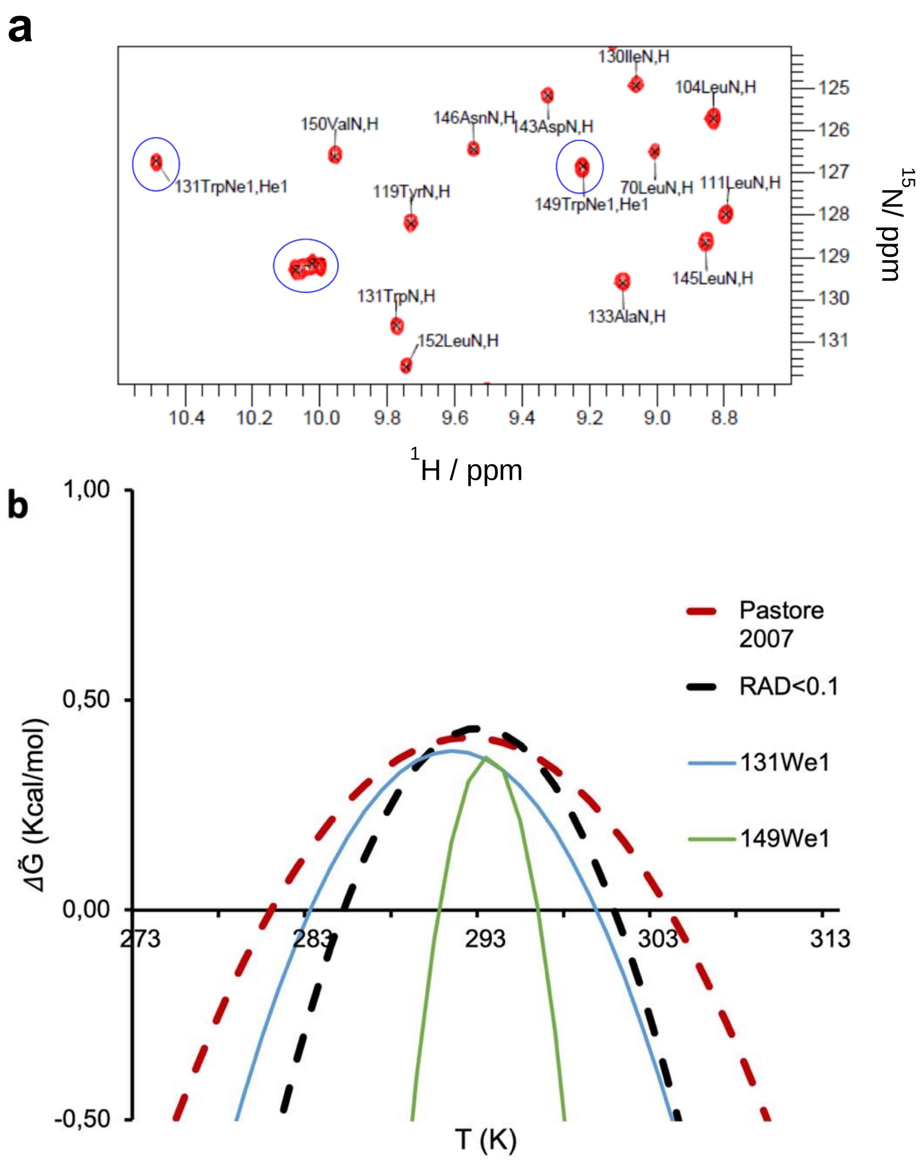

Figure S5. Unfolding based on tryptophan side chains. **a)** Enlarged region of the ^{15}N HSQC spectrum of Yfh1 illustrating the environment of the side chain NHs. The three adjacent peaks at ca.10.05 and 128.2 ppm (circled) disappear from the spectrum when salt is added (Vilanova et al., 2014). They were thus assigned to belong to unfolding intermediates of W149. **b)** Comparison of the stability curve derived from the NE1 resonance of W131 (green) with that of RAD_0.1. The curve for W149 NE1 (orange) reported for comparison, is characterized by an impossibly high value of ΔCp.

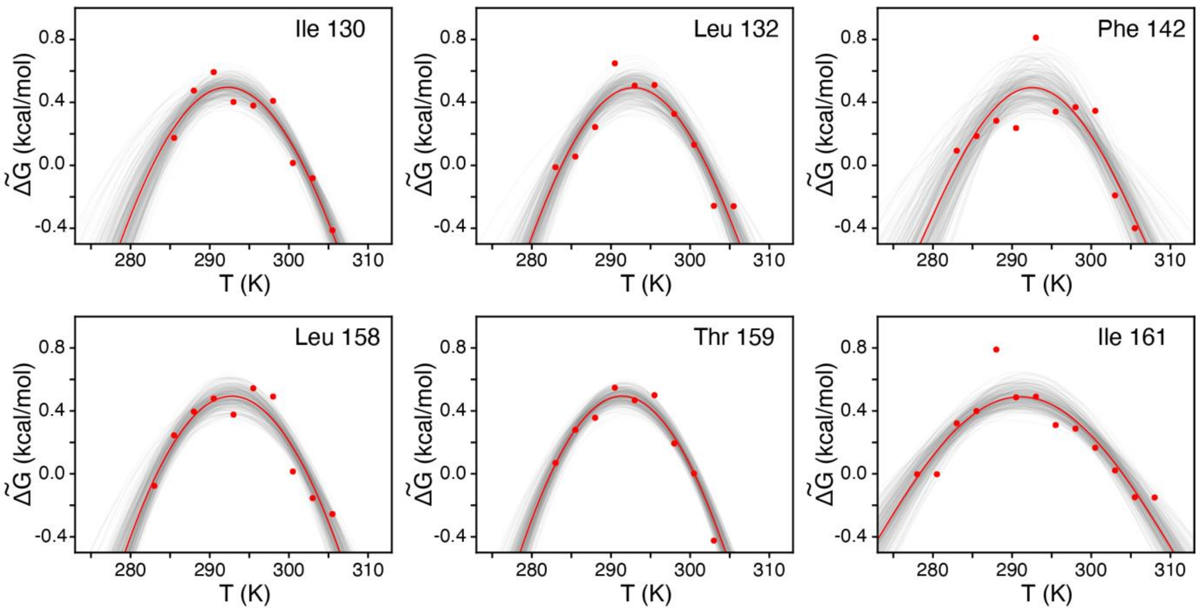

Figure S6. Representative stability curves of residues belonging to the RAD_0.1 set. Red circles are per-residue ΔG values derived from the populations; vertical red lines are estimated errors on ΔG values. Black solid lines represent the best least-squares fit of the equation for the stability curve to the obtained ΔG; Grey lines are the confidence interval, represented by 250 randomly sampled curves from the covariance matrix (multi-variate normal distribution).

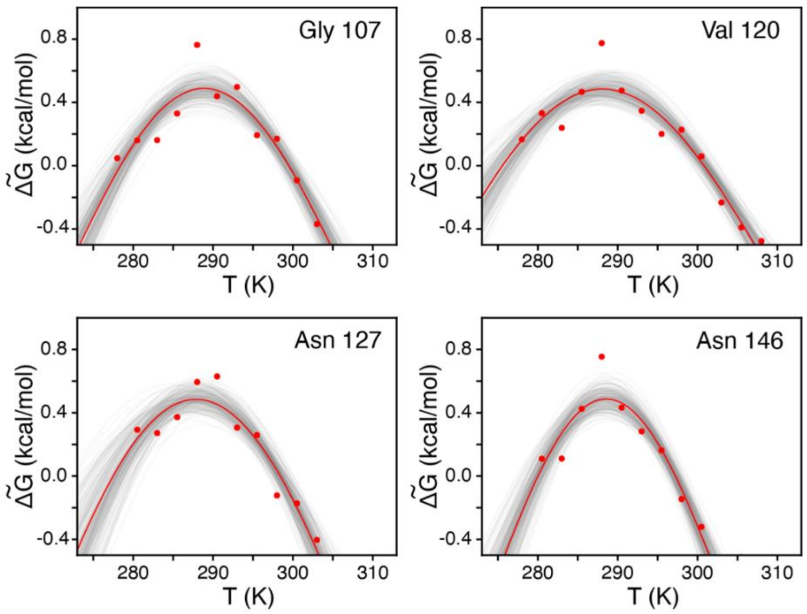

Figure S7. Stability curves of representative residues from **Figure 1c**. Red circles are per-residue ΔG values derived from the populations; vertical red lines are estimated error in ΔG. Black solid line represents the best least-squares fit of the equation for the stability curve to the obtained ΔG; Grey lines are the confidence interval, represented by 250 randomly sampled curves from the covariance matrix (multi-variate normal distribution).

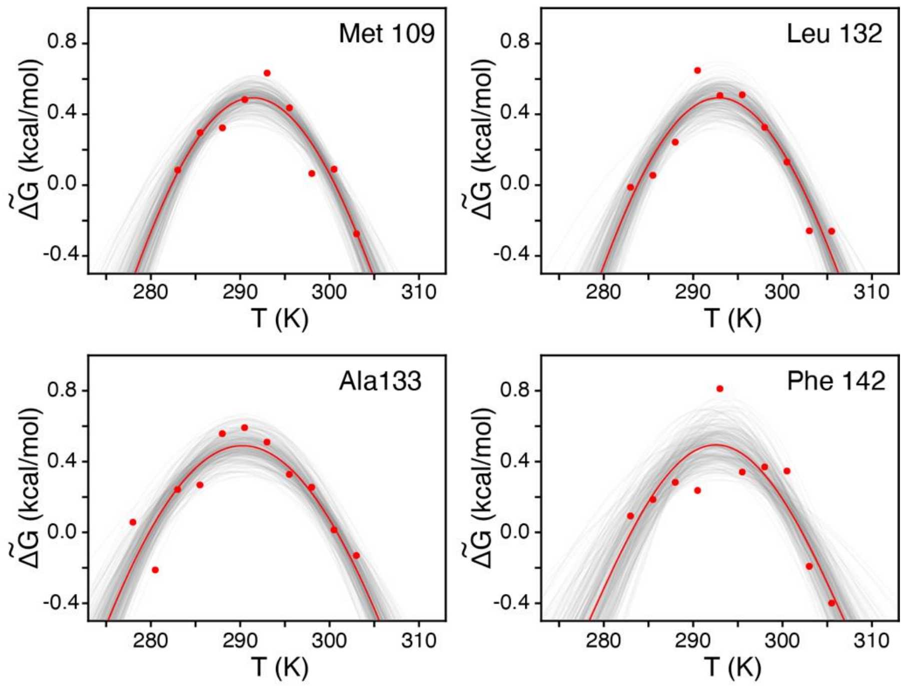

Figure S8. Stability curves of some of the best residues of the β-strands (see Figure 5). Red circles are per-residue ΔG values derived from the populations; vertical red lines are estimated error in ΔG. Black solid line represents the best least-squares fit of the equation for the stability curve to the obtained ΔG; Grey lines are the confidence interval, represented by 250 randomly sampled curves from the covariance matrix (multi-variate normal distribution).

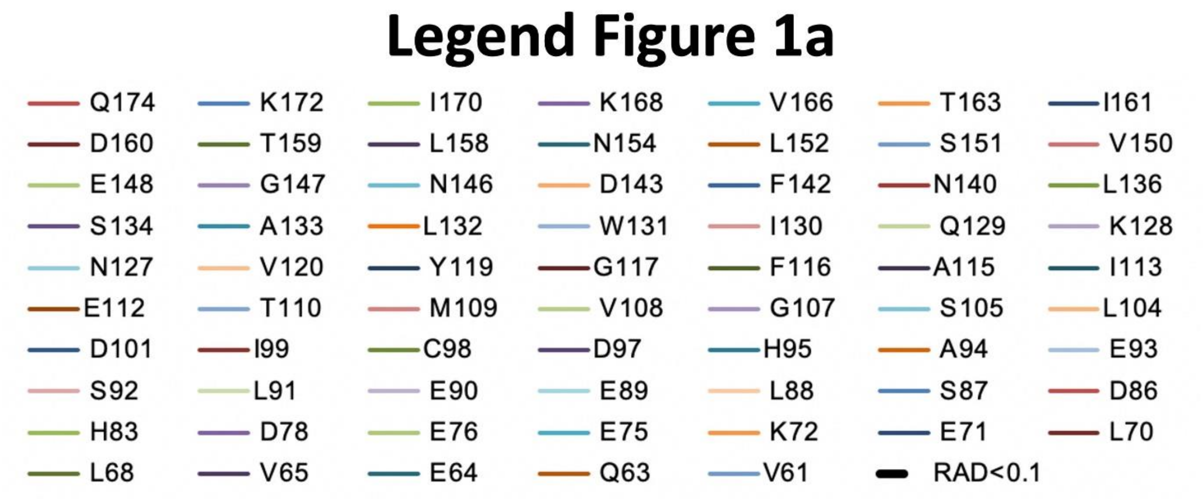

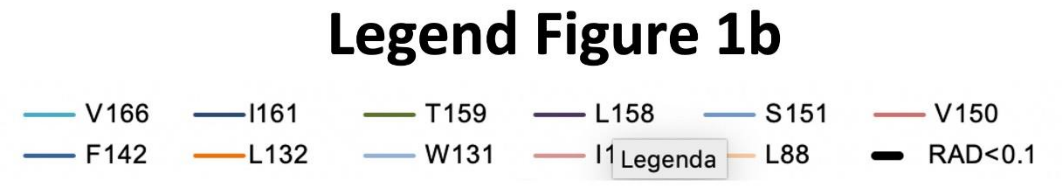

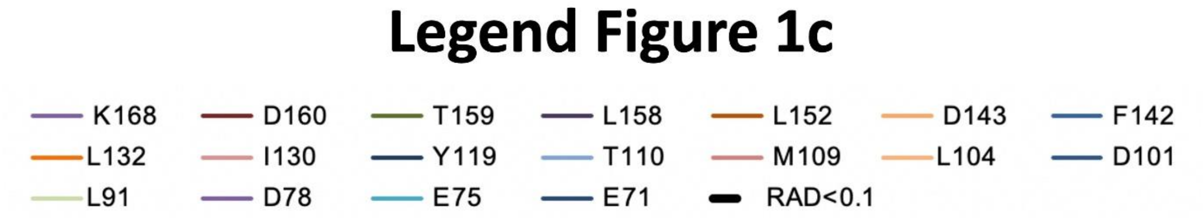

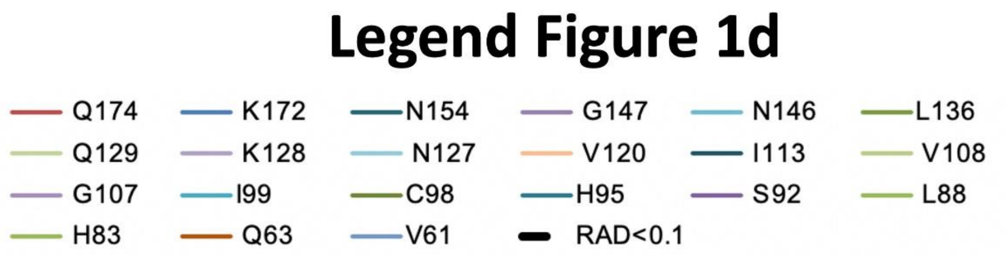

Table S1. Color coding used in Figure 1 of the main text.
